# An Ultrasound–Fenton Process for the Degradation of 2,4,6-Trinitrotoluene

**DOI:** 10.3390/ijerph20043102

**Published:** 2023-02-10

**Authors:** Yangang Li, Wenzhen Zhang, Kelei Mu, Shangkun Li, Jiawei Wang, Shujun Zhang, Lu Wang

**Affiliations:** 1 Research and Development Center, Beijing Drainage Group Co., Ltd., Beijing 100044, China; 2 Laoshan Laboratory, Qingdao 266237, China

**Keywords:** 2,4,6-TNT, ultrasound-Fenton (US–Fenton) processes, removal efficiency, TNT degradation pathway

## Abstract

2,4,6-Trinitrotoluene (TNT), one of the main compounds in ammunition wastewater, is harmful to the environment. In this study, the treatment efficiency of 2,4,6-TNT by different treatment processes, including ferrous ion (Fe^2+^), hydrogen peroxide (H_2_O_2_), Fenton, ultrasound (US) irradiation, US + Fe^2+^, US + H_2_O_2_ and US–Fenton process, was compared. The results showed that US–Fenton was the most effective among all methods studied. The effects of initial pH, reaction time and H_2_O_2_ to Fe^2+^ molar ratio were investigated. The results showed that the removal of TNT, TOC and COD was maximum at an initial pH of 3.0 and H_2_O_2_ to Fe^2+^ molar ratio of 10:1. TNT, TOC and COD removal was fast in the first 30 min, reaching 83%, 57% and 50%, then increased gradually to 99%, 67% and 87% until 300 min, respectively. Semi-batch mode operation increased the removal of TNT and TOC by approximately 5% and 10% at 60 min, respectively. The average carbon oxidation number (ACON) was increased from −1.7 at 30 min to a steady-state value of 0.4, indicating the mineralization of TNT. Based on GC-MS analysis, 1,3,5-trinitrobenzene, 2,4,6-trinitrobenzene acid, 3,5-dinitrobenznamine and 3,5-dinitro-p-toluidine were the major byproducts from the US–Fenton process. The TNT degradation pathway was proposed, which involved methyl group oxidation, decarboxylation, aromatic ring cleavage and hydrolysis.

## 1. Introduction

2,4,6-trinitrotoluene (TNT), one of the priority compounds listed by the United States Environmental Protection Agency (U.S. EPA), is one of the most widely used nitroaromatic explosives, which is also known for its mutagenic potency [1]. Rodgers and Bunce reported that the TNT concentration in contaminated soil and groundwater sites could reach 10 to 1200 ppm [2]. During World Wars I and II, fatal cases of toxic jaundice and aplastic anemia were recorded among munitions workers [3]. In order to protect human health, the U.S. EPA established a rigorous ambient criterion of 0.06 mg/L for TNT and the TNT limit in drinking water is 0.049 mg/L [4].

In order to treat TNT-contaminated soils and waters, various conventional physical (e.g., activated carbon absorption), chemical (e.g., birnessite reduction) and biological (e.g., aerobic biodegradation by *Bacillus cereus*) methods have been investigated [5,6,7]. Although these methods could remove TNT to some extent, there were also some disadvantages, such as high treatment cost, need of additional ex situ treatment [8] and low removal efficiency [9]. Recently, there is considerable focus on advanced oxidation processes (AOPs), mainly utilizing hydroxyl radical (HO^•^) as an oxidant, for the treatment of TNT-contaminated waters, which can lead to less-harmful biodegradable compounds or complete mineralization. For instance, formic and acetic acids, NO_3_^−^, CO_2_ and H_2_O could be the final products of TNT degradation.

The Fenton process and ultrasonic irradiation (US) are two typical AOP methods, which have been used to treat various types of wastewaters [10,11,12,13,14,15,16,17]. The Fenton process is based on an electron transfer between hydrogen peroxide (H_2_O_2_) and ferrous (Fe^2+^), as shown in Equations (1) and (2).

H_2_O_2_ + Fe^2+^ → Fe^3+^ + OH^−^ + HO^•^ (1)

H_2_O_2_ + Fe^3+^ → Fe^2+^ + HO_2_^•^ + H^+^ (2)

Different from many other radicals, HO^•^ can readily attack a large group of organic and inorganic chemicals non-selectively and convert them into less complex and harmful intermediates or products. Ultrasonic irradiation is also a promising technology for decomposing recalcitrant chemicals. During ultrasonication, the transient collapse of cavitation bubbles can create an energetic micro-environment of extremely high local temperature (4000–5000 K) and pressure (up to 5000 atm) [18]. As shown in Equations (3)–(5), the thermolytic decomposition of bubble contents in the micro-environment can generate free radical species (HO^•^, H^•^ and HO_2_^•^) [19], which renders dissolved solutes decomposed or mineralized at the gas–liquid interface or in the bulk liquid.

H_2_O + ))) → H^•^ + HO^•^ (3)

HO^•^ + HO^•^ → H_2_^−^ + O_2_ (4)

H^•^ + O_2_ → H_2_O^•^ (5)

The ultrasound–Fenton (US–Fenton) process is a complex AOP reaction system and has shown several advantages on the treatment of recalcitrant contaminants. Bansturk et al. [20], Grcic et al. [21] and Segura et al. [22] studied the treatment of organic wastewaters using the US–Fenton process and found that the treatment efficiency of the US–Fenton surpassed that of the individual processes of US or Fenton. The synergistic effects of the combined US–Fenton process enhanced treatment performance. Furthermore, other functions of ultrasonic irradiation, such as thermal effects and mechanical actions, inhibited the formation of ferric hydroxide and ferric complexes that occurred in the Fenton reaction, which could also increase the treatment efficiency of the US–Fenton process. At the same time, some free radicals generated by US can oxidize or reduce the functional groups of organic compounds and, thereby, enhance the degradation of organic chemicals. However, to the best of our knowledge, there has no previous research on the degradation of TNT by the combined US–Fenton process.

The objectives of the present study were: (1) to determine the optimal process conditions for the TNT wastewater treatment by the US–Fenton process, (2) to identify the main degradation intermediates, and (3) to establish the TNT degradation pathways.

## 2. Materials and Methods

### 2.1. Materials

Analytical TNT (30 wt%) was obtained from Chem-Service (West Chester, PA, USA). The standard TNT solution (1000 µg/mL in acetonitrile), ferrous sulfate (FeSO_4_•7H_2_O, 95%), hydrogen peroxide (H_2_O_2_, 30 wt%) and methanol (HPLC grade, 99.9%) were provided by Fisher Scientific (Pittsburgh, PA, USA).

### 2.2. Experimental Process

Three series of experiments were carried out utilizing a borosilicate glass vial (working volume 40 mL) as the reactor, and the experimental conditions are listed in Table 1. Groups I–VII were conducted to compare the TNT degradation performance of 7 treatment processes, including Fe^2+^, H_2_O_2_, Fenton, ultrasound (US) irradiation, US + Fe US + H_2_O_2_ and the combination of US and Fenton (US–Fenton). The optimal treatment process was screened in terms of the TNT and TOC removal efficiencies. Subsequently, the effects of initial pH (Group VIII), molar ratio of H_2_O_2_ to Fe^2+^ (Group IX), reaction time (Group X) and dosing mode of Fenton reagents (Groups XI and XII) on the TNT degradation were investigated to optimize the operational conditions of the US–Fenton process. Group XIII was performed to determine the nitrogen mass balance and the TNT degradation pathway during the US–Fenton treatment process.

For all the experimental groups, the reactor was filled with 30 mL of TNT solution, with an initial concentration of 30 mg/L and pH of 3.0 ± 0.1. The ionic strength and the initial pH were adjusted to 0.01 M and the desired values in Table 1, respectively, according to the reported procedure [23]. The reaction temperature was maintained at 25 °C with a temperature control system (Frigomix 1495, Fisher Scientific) coupled with a water circulation apparatus. US radiation was provided by a US generator (Cole-Parmer 600-Watt, 20 kHz) and the titanium probe (Cole Parmer, Model CV 17) was inserted into the reactor to initiate the reaction. Group XII was operated in semi-batch mode, i.e., the Fenton reagents were continuously dosed into the reactor at a certain flow rate (Table 1) using a peristaltic pump (Masterflex, Model 77120-62, flow range: 0.002–12.3 mL/min). However, the other experimental groups were operated in batch mode, i.e., the Fenton reagents were instantaneously spiked into the reactor. At a pre-selected reaction time, water samples were taken from the reactor, immediately treated by Manganese dioxide to stop the Fenton reaction and then subjected to the analysis of residual TNT, TOC, COD, soluble iron, H_2_O_2_, hydrocarbon intermediates, nitrate and nitrite. All experiments were performed in duplicate.

### 2.3. Analytical Methods

The TNT concentration was monitored using a Perkin Elmer high-performance liquid chromatography (HPLC) system equipped with a Jasco 875 UV/VIS detector (λ = 240 nm) and a Luna C-18 column (150 × 2 mm, Phenomenex). The column was maintained at 30 °C and the injection volume was 100 μL. The mixture of 30% water and 70% methanol (*v*/*v*) was employed as the mobile phase with a flow rate of 0.15 mL/min. The intermediates of TNT degradation were analyzed using a Gas Chromatograph–Mass Spectrometer (GC/MS). The treated sample was extracted and then the extracts were analyzed by GC/MS. The detailed extraction procedure is presented in Supporting Information Section S1. The oven was programmed from 70 to 200 °C for 4 °C min^−1^. Helium was used as the carrier gas at a flow rate of 0.6 mL/min. Detection was achieved through flame ionization maintained at 300 °C.

Chemical oxygen demand (COD), residual H_2_O_2_, total organic carbon (TOC), soluble ferrous iron and total soluble iron were measured following the methods reported in our previous research [23]. Nitrite and nitrate were analyzed with an HPLC equipped with a Jasco 875 UV/VIS detector (λ = 210 nm) and a Luna C-18 column (150 × 2 mm, Phenomenex). The column was maintained at 40 °C and the injection volume was 5 μL. The mixture of n-octylamine (0.01 M, pH = 4) and tetrabutylammonium hydrogen sulphate (5 mM, pH = 6.5) served as the mobile phase at a flow rate of 1.0 mL min^−1^. Ammonia concentration was determined by a 4-Star pH/ISE meter with an ammonia ion-selective electrode.

## 3. Results and Discussion

### 3.1. Screening of Various Treatment Processes

**Figure 1:**
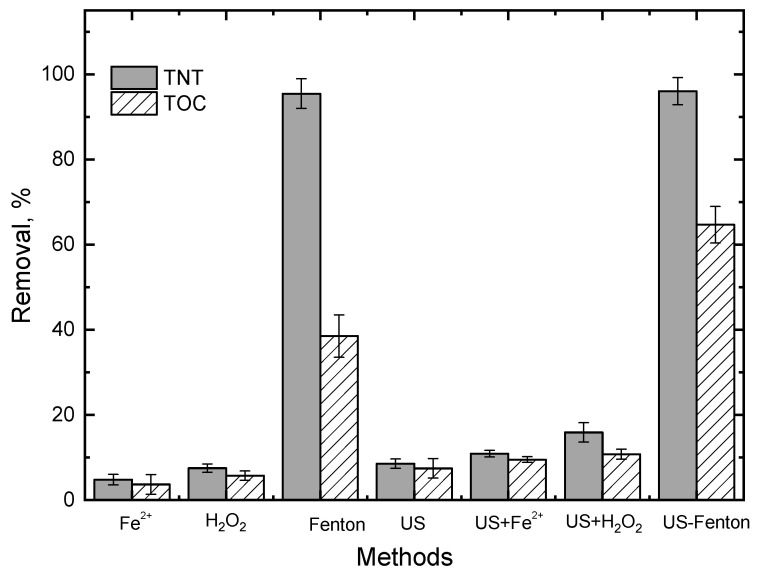
TNT and TOC removal by different AOP methods. Experimental conditions: (TNT)_0_ = 30 mg/L, Initial pH = 3.0 ± 0.1, (Fe^2+^) = 5 × 10^−4^ M, (H_2_O_2_) = 5 × 10^−3^ M, (NaCl) = 10^−2^ M, US intensity = 8571 W/m^3^, Temperature = 25 °C, Reaction time = 60 min, US: ultrasound irradiation, US–Fenton: ultrasound–Fenton process.

Figure 1 and Table S1 show the removal of TNT and TOC via seven treatment processes. The results indicated that Fe^2+^, H_2_O_2_, US, US + Fe^2+^ and US + H_2_O_2_ had low removal efficiencies of TNT (<20%) and TOC (<15%) in 60 min. The TNT removal efficiency of Fenton (>95%) was close to that of US–Fenton (>96%), while the TOC removal efficiency of Fenton (38%) was significantly lower than that of US–Fenton (62%). Therefore, the parent TNT compound was readily degradable, but the intermediates were somewhat difficult to completely mineralize. Chen et al. [24] reported that HO^•^ oxidized the methyl group of TNT to 1,3,5-trinitrobenzene (TNB) (Equation (6)), which was more stable than the parent TNT during the Fenton process. Furthermore, the presence of US facilitates the Fenton reaction due, in part, to the generation of H^•^ radical under US irradiation. H^•^ radical could readily react with the nitro groups of the organic compound, which enhanced the overall degradation of TNT. Cavitation is another contribution force enhancing the combustion of TNT and its intermediates trapped inside the microbubbles during the US–Fenton treatment.

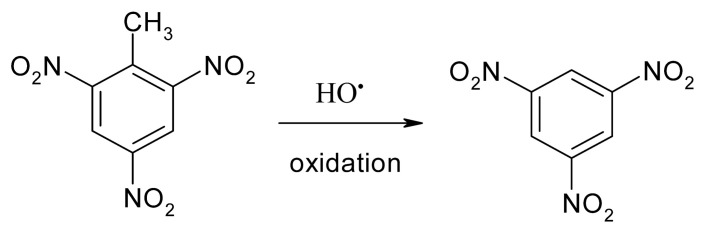
(6)

One-way ANOVA analysis was used to determine whether there were significant differences in removal performance among these treatment processes. The results showed that there were no significant differences in the TNT removal efficiencies among Fe^2+^, H_2_O_2_ and US (*p* > 0.05), while a significant difference was observed between any two processes in US, US + Fe^2+^, US + H_2_O_2_ and Fenton (*p* < 0.05). Additionally, US–Fenton and Fenton showed little difference (*p* > 0.05). Therefore, the TNT removal efficiencies of these treatment processes followed an order: US–Fenton ≈ Fenton > US + H_2_O_2_ > US + Fe^2+^ > US ≈ H_2_O_2_ ≈ Fe^2+^, which was also appropriate for the TOC removal efficiencies of the seven processes. The US–Fenton process had significant superiority over the other studied treatment processes.

### 3.2. Effect of Initial pH on TNT Degradation

The US–Fenton process was then studied further to identify the major operation parameters on its TNT treatment efficiency.

**Figure 2:**
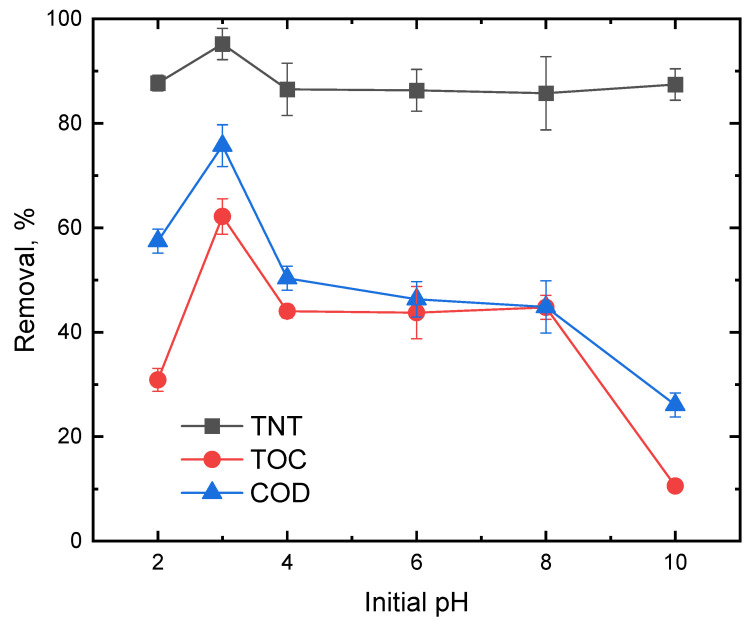
Effect of pH on TNT, TOC and COD removals. Experimental conditions: (TNT)_0_ = 30 mg/L, (Fe^2+^) = 5 × 10^−4^ M, (H_2_O_2_) = 5 × 10^−3^ M, (NaCl) = 10^−2^ M, US intensity = 8571 W/m^3^, Temperature = 25 °C, Reaction time = 60 min.

First, the effect of initial pH (i.e., 2, 3, 4, 6, 8 and 10) on the removal of TNT, TOC and COD was studied (Figure 2). The removal of TNT, COD and TOC was optimal at an initial pH of 3.0, which was in agreement with Cui et al. [25], who studied the treatment of nonylphenol ethoxylates (NPEOs) via the Fenton oxidation process in an aqueous solution and reported an optimal initial pH of 3. HO^•^ was the main oxidizing reagent in the Fenton process. At pH = 2, generated HO^•^ was scavenged by excessive H^+^ in the solution. As pH deceased below 2, the formation of (Fe (H_2_O)_6_)^2+^ retarded the process of Fe^2+^ reacting with H_2_O_2_ to produce HO^•^, which was much less reactive with hydrogen peroxide [26]. In addition, the proton reacted with H_2_O_2_ to form an oxonium ion (H_3_O_2_^+^) (Equation (7)), which rendered H_2_O_2_ electrophilic and enhanced its stability and decreased the reactivity between H_2_O_2_ and Fe^2+^ [14]. Therefore, low pH did not favor TNT degradation.

H_2_O_2_ + H^+^ → H_3_O_2_^+^ (7)

Under high pH conditions (i.e., pH ≥ 10), the removal of TOC and COD was low due to the decrease in HO^•^ production. In the Fenton reaction, high pH might contribute to the formation of ferrous and ferric hydroxyl complexes, which might lead to a decrease in the production of HO^•^. Furthermore, at pH > 4, both Fe^2+^ and Fe^3+^ were precipitated as iron hydroxides, which decreased the concentration of free iron needed for the Fenton reaction. The decrease in the oxidation potential of HO^•^ at a high pH might also contribute to the decrease in TOC and COD removal [16]. At pH > 8–10, TNT removal remained high at ca. 85%, while TOC and COD removal was ca. <10–40%, indicating that TNT could be effectively decomposed under high pH but only a small fraction of parent TNT could be mineralized. That is because high pH could significantly decrease the concentration of dissolved iron and, thereby, lead to less generation of the hydroxyl radical.

### 3.3. Effect of H_2_O_2_ to Fe^2+^ Molar Ratio

**Figure 3:**
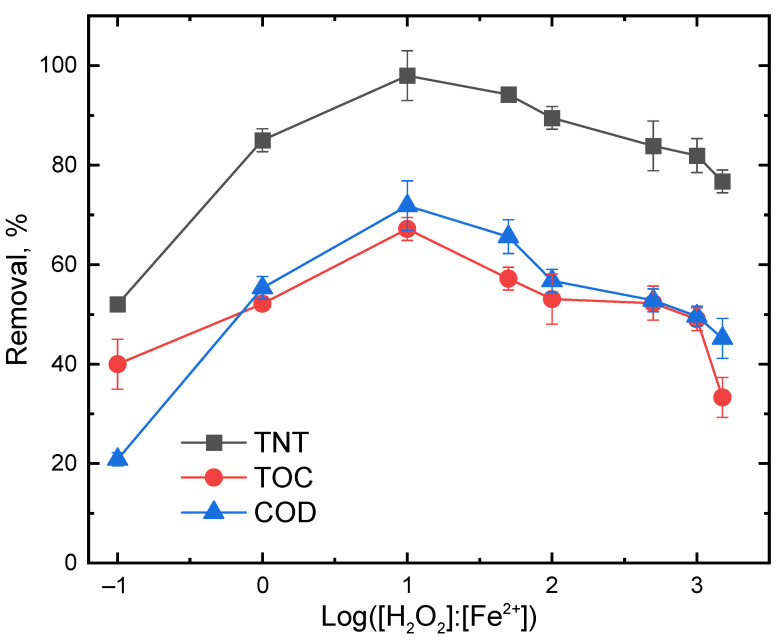
Effect of H_2_O_2_ to Fe^2+^ molar ratio on the removal of TNT, TOC and COD. Experimental conditions: (TNT)_0_ = 30 mg/L, (Fe^2+^) = 5 × 10^−4^ M, Initial pH = 3.0 ± 0.1, (NaCl) = 10^−2^ M, US intensity = 8571 W/m^3^, Temperature = 25 °C, Reaction time = 60 min.

Figure 3 shows the effect of H_2_O_2_ to Fe^2+^ molar ratio on TNT degradation by the US–Fenton process. To determine the optimal molar ratio, the ferrous iron dose was kept constant at 28 mg/L (or 5 × 10^−4^ M) and the H_2_O_2_ concentration was varied to yield H_2_O_2_ to Fe^2+^ molar ratio in a range from 0.1 to 1500. The removal of TNT, TOC and COD increased quickly as the molar ratio increased from 0.1 to 10; afterwards, a slow decrease in the TNT, TOC and COD removal was observed as the molar ratio further increased to 1500. Therefore, the highest removal of TNT, TOC and COD (i.e., 98%, 67% and 72%) was reached at a molar ratio of 10. Our results were in agreement with results from Cui et al. [26], who reported that the decomposition of nonylphenol ethoxylates (NPEOs) was increased when the H_2_O_2_ to Fe^2+^ molar ratio was increased from 1 to 4 and then decreased when the molar ratio was increased from 4 to 4.5. A low molar ratio led to excessive Fe^2+^ to react with HO^•^, decreasing the US–Fenton’s efficiency (Equation (8)), whereas a high H_2_O_2_ to Fe^2+^ molar ratio enhanced the consumption of HO^•^ by the excess H_2_O_2_ by transforming HO^•^ to HO_2_^•^ (Equation (9)), which has a lower oxidation potential than HO^•^. Furthermore, the incremental generation of HO_2_^•^ could also be consumed by HO^•^ according to Equation (10). Nam et al. [27] and Zhang et al. [28] reported that the optimum H_2_O_2_ to Fe^2+^ molar ratio was dependent on the type, concentration and the mineral contents of the wastewaters.

Fe^2+^ + HO^•^ → Fe^3+^ + OH^−^ (8)

H_2_O_2_ + HO^•^ → HO_2_^•^ + H_2_O (9)

HO_2_^•^ + HO^•^ → O_2_ + H_2_O (10)

### 3.4. Effect of Reaction Time

**Figure 4:**
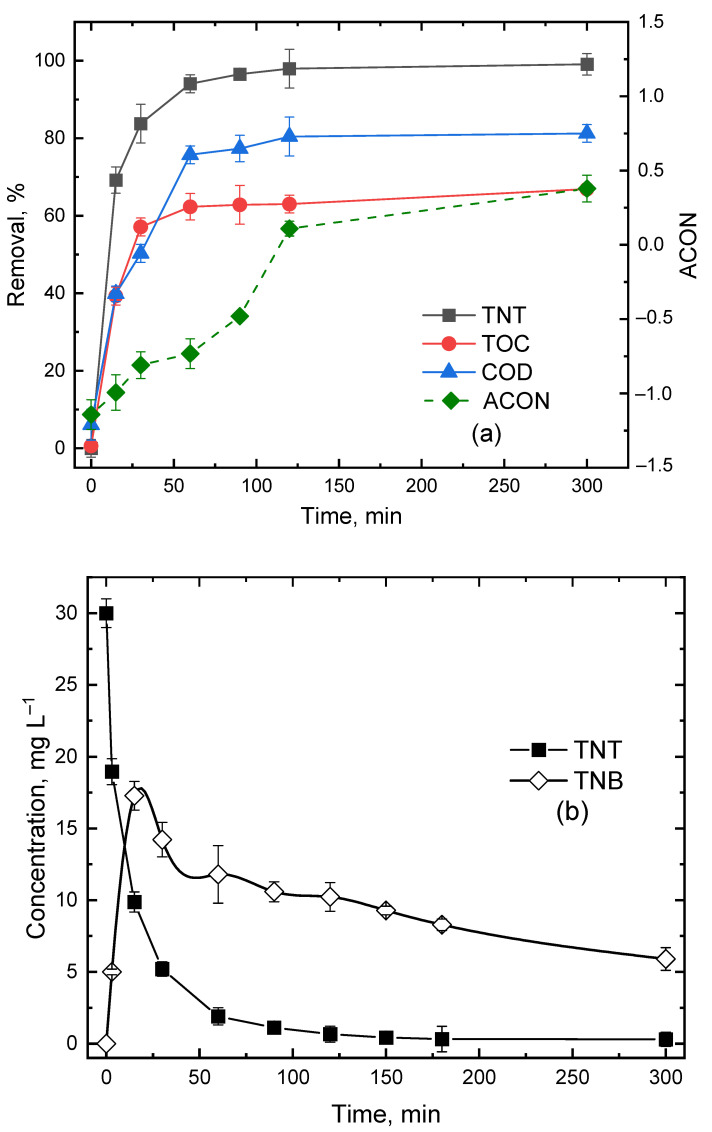
Effect of reaction time on the removal of TNT, TOC and COD (**a**) and change in TNT and TNB concentration as a function of time (**b**). Experimental conditions: (TNT)_0_ = 30 mg/L, (Fe^2+^) = 5 × 10^−4^ M, (H_2_O_2_) = 5 × 10^−3^ M, (NaCl) = 10^−2^ M, Initial pH = 3.0 ± 0.1, US intensity = 8571 W/m^3^ Temperature = 25 °C, Reaction time = 300 min.

Figure 4a shows the removal of TNT, TOC and COD as a function of reaction time. The TNT, TOC and COD removal was increased rapidly in the first 60 min, reaching 94, 62 and 75%, respectively. For the remaining 240 min until 300 min, TNT, TOC and COD removal was slowly reaching steady-state values of 99, 67 and 87%, respectively. Generally, percent TNT removal was higher than that of COD and TOC, due probably to the direct oxidation of the methyl group by HO^•^ to carboxyl or decarboxylase and to TNB or other organic compounds, as shown in Figure 4b. In contrast, the removal of TOC and COD was a complex process. According to Lyman et al. [29], the degradation of organic compounds by Fenton’s reagent could be divided into three distinct stages: primary, intermediate and ultimate. Primary and intermediate TOC degradation changed the structure of the parent compound and, thus, reduced toxicity, while ultimate TOC degradation resulted in complete mineralization of organic compounds to carbon dioxide, water and other inorganics. The degradation of TNT happened largely in the primary stage, but COD changed accordingly during the TOC degradation stage. Therefore, the TNT removal percent was higher than TOC and COD. Moreover, TNB as the main intermediate was relatively stable and had a residual concentration of 8 mg/L after 5 h of US–Fenton treatment (Figure 4b), resulting in a reduction in TOC and COD removal in comparison to TNT. In order to understand the degree of carbon mineralization of the parent organic compound, the average carbon oxidation number (ACON) was determined, which can be calculated by the following equation [30]:

(11) $$\mathrm{ACON}=\frac{4\left(\mathrm{TOC}-\mathrm{COD}\right)}{\mathrm{TOC}}$$

Figure 4a shows that the ACON was increased from −1.1 to 0.4 over the time period of 300 min. Note that a larger positive ACON is indicative of a higher degree of mineralization. The persistent nature of the intermediates might prevent further oxidization to inorganic carbonates; therefore, it would be rather difficult to achieve 100% COD and TOC removal [17,31].

### 3.5. TNT Degradation in Semi-Batch Mode

**Figure 5:**
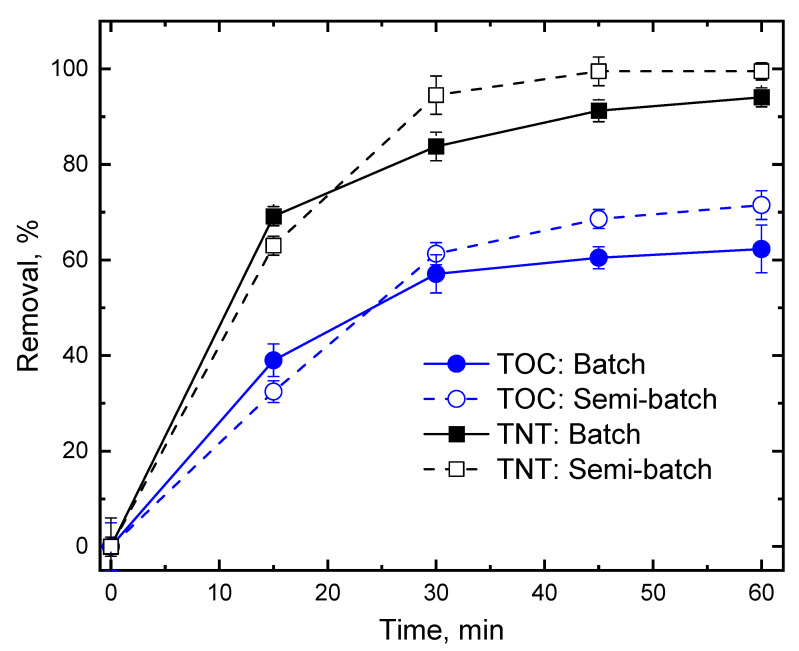
Removal of TNT and TOC by batch and semi-batch mode reactor. Experimental condition: (TNT)_0_ = 30 mg/L, (NaCl) = 10^−2^ M, Initial pH = 3.0 ± 0.1, Temperature = 25 °C, US intensity = 8571 W/m^3^, Reaction time = 60 min, Batch: (Fe^2+^) = 5 × 10^−4^ M, (H_2_O_2_) = 5 × 10^−3^ M, Semi-batch: Dosing rate of Fenton reagents: (H_2_O_2_) = 2.5 × 10^−6^ mol/min, (Fe^2+^) = 2.5 × 10^−7^ mol/min.

In order to investigate the effect of the dosing strategy of Fenton reagents on treatment efficiency, semi-batch experiments were conducted by continuingly dosing both H_2_O_2_ and Fe^2+^ (Figure 5). At the onset of the experiment, both TNT and TOC removals were enhanced slightly in the batch compared to the semi-batch experiment. At about 25 min, the removal of TNT and TOC became higher in semi-batch than in batch mode. At the end of the experiment, i.e., 60 min, the removal of TNT was 100% in semi-batch mode compared to 90% in the batch reactor. Similar to TNT removal, semi-batch mode removed 10% more TOC than the batch experiment. Because Fenton reagents were added in plug to the reaction system once, a large amount of HO^•^ was generated immediately, which degraded TNT and TOC rapidly at the onset of the batch experiment. In comparison, at the beginning of the semi-batch experiment, there was less HO^•^ generation, which resulted in lower TNT and TOC removal. However, in the semi-batch experiment, there was less HO^•^ consumption due to the continuing supply of Fenton reagents, thereby resulting in higher removal of TNT and TOC than the batch mode at the end of the experiments.

**Figure 6:**
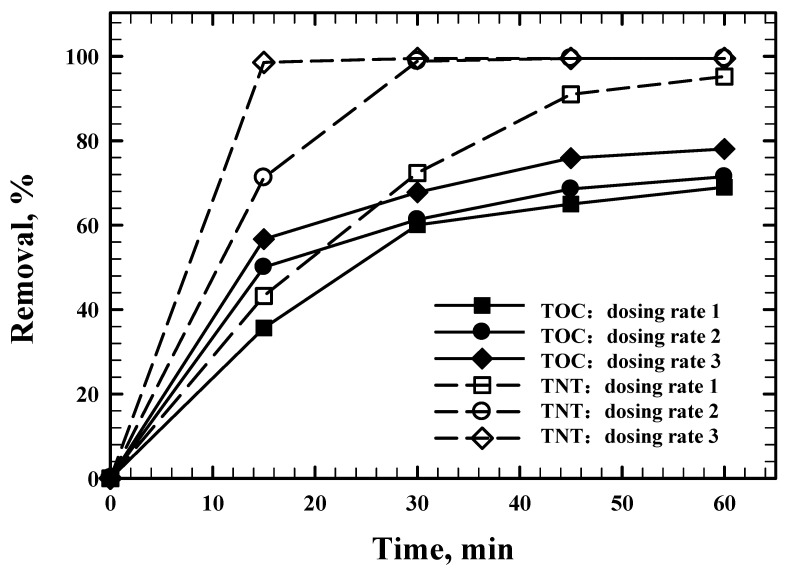
Effect of dosing rate on TNT and TOC removal. Experimental condition: (TNT)_0_ = 30 mg/L, (NaCl) = 10^−2^ M, Initial pH = 3.0 ± 0.1, Temperature = 25 °C, US intensity = 8571 W/m^3^, Reaction time = 60 min, Dosing rate 1: (H_2_O_2_) = 2.5 × 10^−6^ mol/min, (Fe^2+^) = 2.5 × 10^−7^ mol/min, Dosing rate 2: (H_2_O_2_) = 5.0 × 10^−6^ mol/min, (Fe^2+^) = 5.0 × 10^−7^ mol/min, Dosing rate 3: (H_2_O_2_) = 7.5 × 10^−6^ mol/min, (Fe^2+^) = 7.5 × 10^−7^ mol/min.

Results in Figure 6 show that an increase in the dosing rate increased TNT and TOC removal. The generation of HO^•^ played a significant role in TNT and TOC removal [32]. Therefore, a higher dosing rate generated a greater amount of HO^•^, which led to greater TNT and TOC removal. The TNT degradation kinetic models with three dosing rates were determined, which followed the pseudo-first-order model. The rate constants under three dosing rates were 0.0412 min^−1^ (R^2^ = 0.996), 0.0925 min^−1^ (R^2^ = 0.946) and 0.1771 min^−1^ (R^2^ = 0.939) for dosing rates 1, 2 and 3, respectively. A higher dosing rate led to a higher TNT degradation rate, which means that the same TNT removal efficiency was achieved in a shorter time with a higher Fenton reagent dosing rate (e.g., 60 min for dosing rates 1 and 30 min for dosing rate 2, and 20 min for dosing rate 3).

### 3.6. Nitrogen Mass Balance

**Figure 7:**
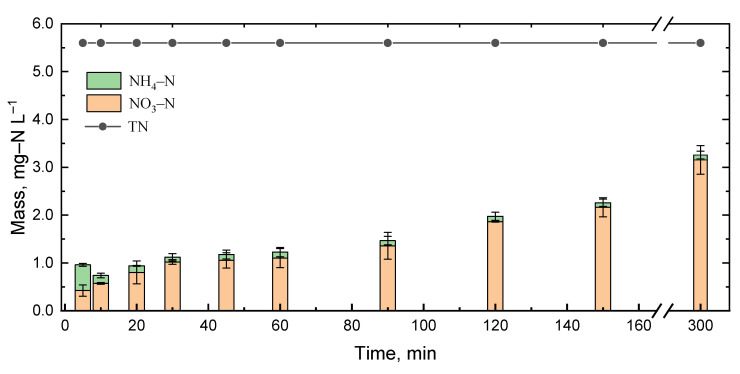
N-mass balance of TNT degradation process. Experimental conditions: (TNT)_0_ = 30 mg/L, (Fe^2+^) = 5 × 10^−4^ M, (H_2_O_2_) = 5 × 10^−3^ M, (NaCl) = 10^−2^ M, Initial pH = 3.0 ± 0.1, US intensity = 8571 W/m^3^, Reaction time = 300 min.

In order to establish the mechanism of TNT degradation, nitrogen recovery was observed through the analysis of NO_3_^−^, NO_2_^−^ and NH_4_^+^. Figure 7 shows the generation of NO_3_^−^, NO_2_^−^, NH_4_^+^ and the total theoretical nitrogen. The US–Fenton process treated a solution containing 30 mg/L of TNT (theoretical N concentration = 5.6 mg/L). In the first 5 min, 0.42 mg-N/L of NH_4_^+^ and 0.53 mg-N/L of NO_3_^−^ were observed. A further increase in reaction time produced less NH_4_^+^ and more NO_3_^−^. At end of the 300 min treatment, the amount of NH_4_^+^ and NO_3_^−^ generated was 0.1 mg-N/L and 3.15 mg-N/L, respectively. The NO_2_^−^ concentration was below the detection limit. NH_4_^+^ formation, possibly from the release of amino functional groups of the intermediates, namely, 3,5-dinitro-p-toluidine, 2-methyl-3,5-dinitrobenzenamine and 3,5-dinitrobenzenamine, reduced by H^•^, which was generated by ultrasonic irradiation (Equation (3)). The decrease in NH_4_^+^ at the end of the experiment might be attributed to ultrasonic irradiation instead of the Fenton reaction as well. Oh et al. [33] reported that the Fenton oxidation of NH_4_^+^ did not result in the formation of NO_3_^−^. Results in Figure 7 show that 33 and 0.8% of nitrogen were recovered as NO_3_^−^ and NH_4_^+^, respectively; NO_3_^−^ was the main species of recovered nitrogen. Ayoub et al. [34] reported that NO_3_^−^ was formed from the cleavage of the nitro groups of TNT via HO^•^ oxidation. At 300 min, only 40% of the nitrogen was recovered while 99% of TNT was removed, which indicated the formation of intermediates of the nitro groups and why TOC removal was less than that of TNT.

### 3.7. Degradation Pathways

Results of GC/MS analysis revealed seven main intermediates, identified as 1,3,5-trinitrobenzene (TNB), 2,4,6-benzonic acid (TNBA), 3,5-dinitrobenzenamine, 1-methyl-2,4-dinitrobenzene (2,4-DNT), 2,4,6-trihydroxybenzoic acid, 3,5-dinitro-p-toluidine and 2-methyl-3,5-dinitrobenzenamine (Figure S1).

TNB is a relatively stable product of TNT degradation via the Fenton process [15,35], formed by the oxidation of TNBA. Schmelling and Gray reported that TNT was first oxidized to 2,4,6-trinitrobenzaldehyde and then rapidly converted to TNBA [36]. However, 2,4,6-trinitrobenzaldehyde was not identified in this work. 1-(2,4,6-trihydroxyphenyl)-ethanone was determined as an intermediate and may be another product of 2,4,6-benzonic acid due to the substitution of the nitro group by HO^•^. Since US generated H^•^ radical, which reduced the –NO_2_ functional group to –NH_2_, TNB was transformed into 3,5-dinitrobenzenamine by this mechanism. Similar results were reported by Doppalapudi et al. [37]. Chen and Liang [38] reported that 2,4-DNT and 1-methyl-2,6-dinitrobenzene (2,6-DNT) were the main degradation products of TNT by electrochemical destruction. In the present work, 2,4-DNT was also identified, coming from one nitro group of TNT undergoing denitrification. 3,5-dinitro-p-toluidine and 2-methyl-3,5-dinitrobenzenamine were possibly another two products of direct TNT degradation, identified by GC/MS. According to our results and the previous literature [39,40], the formation of the above intermediates occurred by the H^•^ reduction in TNT.

**Figure 8:**
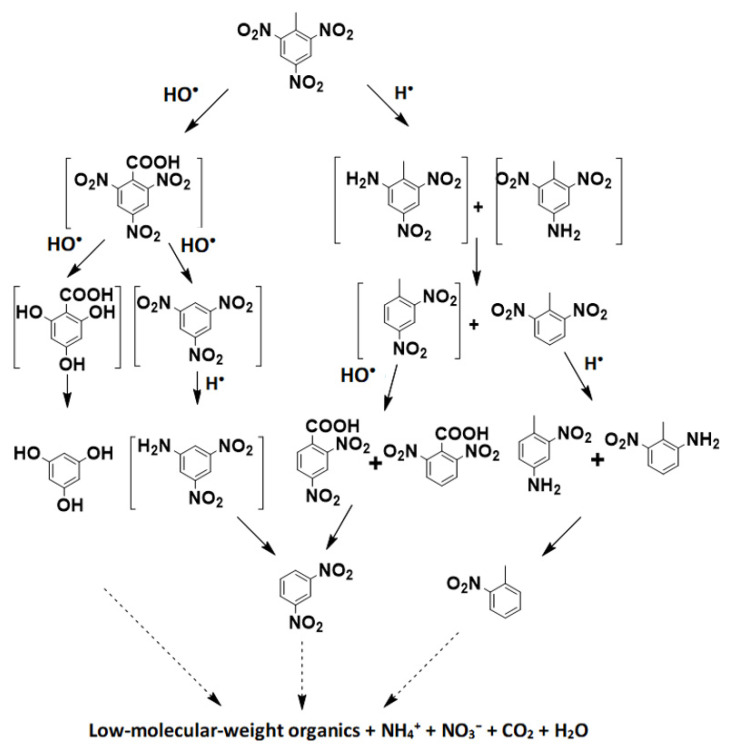
Degradation pathway of TNT by US–Fenton treatment. Organic compounds in square brackets were intermediates identified by GC/MS in this study.

Due to the complexity of the US–Fenton process, physical and chemical reactions occurred simultaneously with the generation of free radicals, such as H^•^ and HO^•^, which brought about hydroxyl addition, hydrogen abstraction, decarboxylation and denitrification. Therefore, two possible mechanisms for the degradation of TNT could be proposed. First, the TNT methyl group was attacked by HO^•^ yielding a TNT radical, which was then oxidized into TNBA. Pyrolytic decarboxylation of TNBA yielded TNB, or substitution of the nitro group by HO^•^ resulted in its conversion to 2,4,6-trihydroxybenzoic acid. Subsequent HO^•^ attack of TNB continuously replaced the nitro group. A further reaction contributed to ring breakage and mineralization to aliphatic organic acids, CO_2_ and H_2_O. Second, the nitro group of TNT was first reduced by a H^•^ to an amine group, which was further oxidized or substituted by a HO^•^, which yielded 1-methyl-2,4-dinitrobenzene and 1-methyl-2,6-dinitrobenzene. Figure 8 illustrates the proposed degradation pathway.

## 4. Conclusions

Different treatment processes, including Fe^2+^, H_2_O_2_, Fenton, US, US + Fe^2+^, US + H_2_O_2_ and US–Fenton process, were screened for TNT degradation. Results revealed that the US–Fenton process was the most effective and was studied further for the effect of initial pH, reaction time and the H_2_O_2_ to Fe^2+^ molar ratio, so as to establish the optimal process parameters. The optimal initial pH and H_2_O_2_ to Fe^2+^ molar ratio was 3.0 and 10:1, respectively. The removal of TNT, TOC and COD was rapid within the first 30 min, reaching 83, 57 and 50%, respectively. Upon a further increase in reaction time, the removal increased gradually to 99% (TNT), 62% (TOC) and 81% (COD) at 300 min. Semi-batch mode experiments increased the TNT and TOC removal by approximately 5% and 10%, respectively. Nitrogen mass balance was determined via the analysis of NO_3_^−,^ NO_2_^−^ and NH_4_^+^ produced. Results demonstrated that 33 and 0.8% of nitrogen were recovered as NO_3_^−^ and NH_4_^+^, respectively, with NO_3_^−^ being the main nitrogen species recovered. Results of GC-MS analysis revealed that 1,3,5-trinitrobenzene, 2,4,6-trinitrobenzene acid, 3,5-dinitrobenznamine, 2-methyl-3,5-dinitrobenzenamine and 3,5-dinitro-p-toluidine were the major intermediates formed from the US–Fenton process. Hence, the TNT degradation pathway was proposed, which involved methyl group oxidation, decarboxylation, aromatic ring cleavage and hydrolysis.

## Figures and Tables

**Table 1:** Design of the experiments.

Group	[Fe^2+^]_0_ (mol/L) ^a^	[H_2_O_2_]_0_ (mol/L) ^c^	[H_2_O_2_]/[Fe^2+^] ^f^	US Intensity (W/m^3^)	Initial pH	Reaction Time (min)
Screening of various treatment processes						
I	5 × 10^−4^	–	–	–	3.0 ± 0.1	60
II	–	5 × 10^−3^	–	–	3.0 ± 0.1	60
III	5 × 10^−4^	5 × 10^−3^	10	–	3.0 ± 0.1	60
IV	–	–	–	8571	3.0 ± 0.1	60
V	5 × 10^−4^	–	–	8571	3.0 ± 0.1	60
VI	–	5 × 10^−3^	–	8571	3.0 ± 0.1	60
VII	5 × 10^−4^	5 × 10^−3^	10	8571	3.0 ± 0.1	60
Influencing factors of TNT degradation by US–Fenton						
VIII	5 × 10^−4^	5 × 10^−3^	10	8571	2.0–10.0 ^h^	60
IX	5 × 10^−4^	5 × 10^−5^–0.75 ^d^	0.1–1500 ^g^	8571	3.0 ± 0.1	60
X	5 × 10^−4^	5 × 10^−3^	10	8571	3.0 ± 0.1	15–300 ^i^
XI	5 × 10^−4^	5 × 10^−3^	10	8571	3.0 ± 0.1	60
XII	2.5 × 10^−7^–7.5 × 10^−7 b^	2.5 × 10^−6^–7.5 × 10^−6 e^	10	8571	3.0 ± 0.1	60
Nitrogen balance and TNT degradation pathways						
XIII	5 × 10^−4^	5 × 10^−3^	10	8571	3.0 ± 0.1	300

^a^ Initial concentration of Fe^2+^. ^b^ The Fe^2+^ was spiked into the reactor with the flow rates of 2.5 × 10^−7^, 5 × 10^−7^, and 7.5 × 10^−7^ mol/min. ^c^ Initial concentration of H_2_O_2_. ^d^ 5 × 10^−5^, 5 × 10^−4^, 5 × 10^−3^, 2.5 × 10^−2^, 0.05, 0.25, 0.50, 0.75 mol/L. ^e^ The H_2_O_2_ was spiked into the reactor with flow rates of 2.5 × 10^−6^, 5 × 10^−6^, 7.5 × 10^−6^ mol/min. ^f^ Molar ratio of H_2_O_2_ to Fe^2+^. ^g^ 0.1, 1, 10, 50, 100, 500, 1000 and 1500. ^h^ 2.0, 3.0, 4.0, 6.0, 8.0 and 10.0. ^i^ 15, 30, 60, 90, 120, 300 min.

## Data Availability

Not applicable.

## Supplementary Materials

The following supporting information can be downloaded at: https://www.mdpi.com/article/10.3390/ijerph20043102/s1, Section S1: Intermediate analysis of TNT degradation by GC/MS; Figure S1: GC/MS profiles of the extract of wastewater treated for different time and the main intermediates; Table S1: Comparison of TNT and TOC removal of various processes. Reference [41] is cited in the supplementary materials.
