# Supplementary material for: An Ultrasound–Fenton Process for the Degradation of 2,4,6-Trinitrotoluene
Source: Int J Environ Res Public Health. 2023 Feb 10;20(4):3102. doi: 10.3390/ijerph20043102 (PMC9961213; doi:10.3390/ijerph20043102)
Supplement: Supplementary file 1 [file ijerph-20-03102-s001.zip › ijerph-2175582-supplementary.pdf]

Supporting Information

An Ultrasound-Fenton Process for the Degradation of 2,4,6-Trinitrotoluene

Yangang Li [1] , Kelei Mu[1], Wenzhen Zhang[1], Shangkun Li[1], Jiawei Wang[1], Shujun Zhang[1]*, and Lu Wang[2]*,

[1] Research and Development Center of Beijing Drainage Group Co., Ltd, Beijing 100044,China

[2] Laoshan Laboratory, Qingdao 266237, China

Corresponding author: * Shujun Zhang (Email: zhangsj@bdc.cn; Tel: +86-10-67758857); Lu Wang (Email: lwang@qnlm.ac)

Table S1. Comparison of TNT and TOC removal of various processes

Treatment schedule	Group number	TNT removal (%)	TOC removal (%)
Fe^{2+}	I	6.80	6.67
H_2O_2	II	7.51	5.74
Fenton	III	96.46	38.53
US	IV	6.57	5.46
US+Fe^{2+}	V	10.89	9.50
US+ H_2O_2	VI	15.89	10.78
US-Fenton	VII	99.04	64.69

S1. 1 Intermediates analysis of TNT degradation by GC/MS

Experimental procedure: 30 mL TNT sample were treated by US-Fenton process. At the predetermined time, 20 mL aliquots of TNT sample were withdrawn for extraction of reaction intermediates.

Experimental conditions: $[TNT]_0$ = 30 mg L^{-1}, [NaCl] = 10^{-2} M, Temperature = 25℃, $[Fe^{2+}]$ = 5×10^{-4}M, $[H_2O_2]$ = 5×10^{-3}M, Initial pH = 3.0 ± 0.1, US intensity = 300 watts cm^{-2}, Reaction time = 0, 10, 30, 60,120 min. In order to stop the Fenton reaction, 1mL 1 M of NaOH was added into the withdrawn sample, and then 20 mL sample was extracted with diethyl ether for intermediate analysis.

Extraction procedure: Aliquots (20 mL) of TNT sample and diethyl ether (2 × 20 mL) were added into a separatory funnel and mixed for 10 min. During shaking release excess pressure. The organic layer was separated from water phase for 15 min. Diethyl ether layer extract was collected in 250 mL Erlenmeyer flask. A second 40 mL diethyl ether and the remaining water phase were added into the separatory funnel and repeated extraction procedure then combined all extracts in the Erlenmeyer flask. The third extraction was performed in the same manner. After that 5 g of anhydrous magnesium sulfate was added to concentrate the extract then filter with 0.45 μm filter. About 1 mL extract was obtained by rotary vapor and collected for GC/MS analysis.

Analysis: Sample extracts were analyzed initially with GC/MS. The oven was programmed from 70 to 200℃ at 4 ℃ min^{-1}. Helium was used as the carrier gas at a flow rate of 0.6 mL min^{-1}. Detection was made by flame ionization, which was maintained at 300 ℃.

Results:

As shown in Figure S1a, there are two compounds in the initial TNT sample, containing Butylated hydroxytoluene (BHT) (Retention time = 26.34 min) and TNT(Retention time = 32.04 min). BHT is an antioxidant. 70% of TNT were degraded when the TNT sample was treated for 10min and one of main intermediates was detected: 1,3,5-trinitrobenzene (Retention time = 31.3 min (Figure S1b). Another two intermediates detected were 2,4,6-benzonic acid (Retention time = 32.84 min) and 3,5-dinitrobenzenamine (Retention time = 42.33 min) when treated for 30 min (Figure S1c). With the reaction time increasing, there was no new main intermediates during the treatment time from 60 to 120 min (Figure S1d-e). Due that Fenton reaction was extremely fast with rate constant as high as 10^7-10^{10} ($M^{-1}sec^{-1}$)[1], some of the intermediates were hardly to be detected. In order to get more information about the degradation pathway, the experiment of TNT treated for 120min by US only was conducted and the intermediates of that were identified. Figure S1f shows the results. Another four intermediates generated were 1-methyl-2,4-dinitrobenzene (Retention time = 27.71 min), 2,4,6-trihydroxybenzoic acid (Retention time = 28.99 min), 3,5-dinitro-p-toluidine (Retention time = 39.81 min), and 2-methyl-3,5-dinitrobenzenamine(Retention time = 42.16 min).

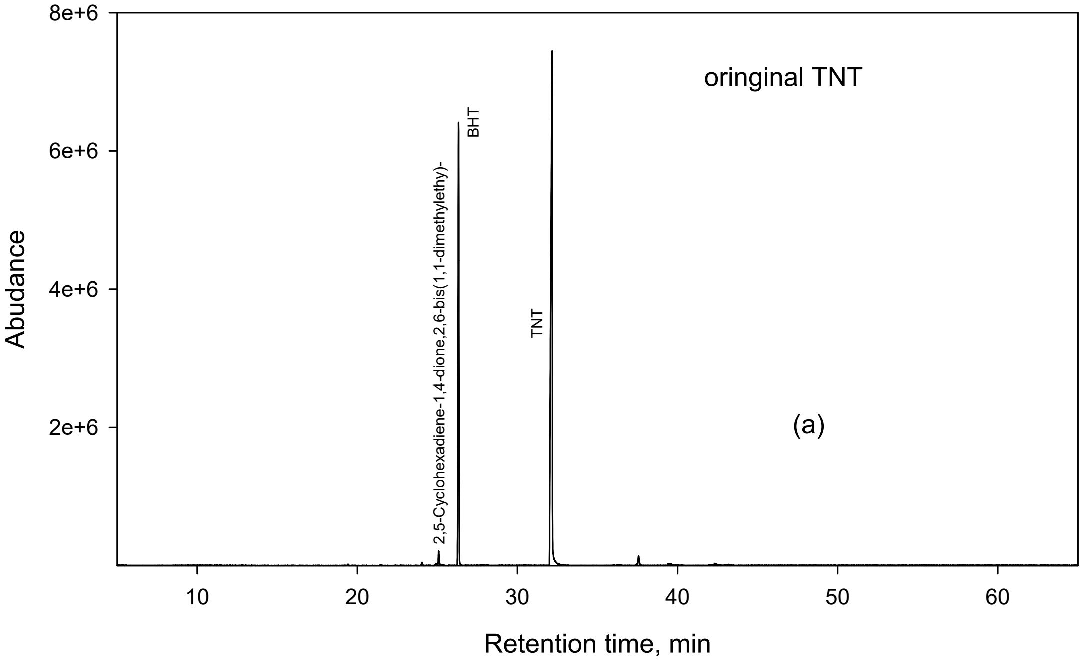
oringinal TNT
(a)
Abudance
Retention time, min
2,5-Cyclohexadiene-1,4-dione,2,6-bis(1,1-dimethylethy)-
BHT
TNT

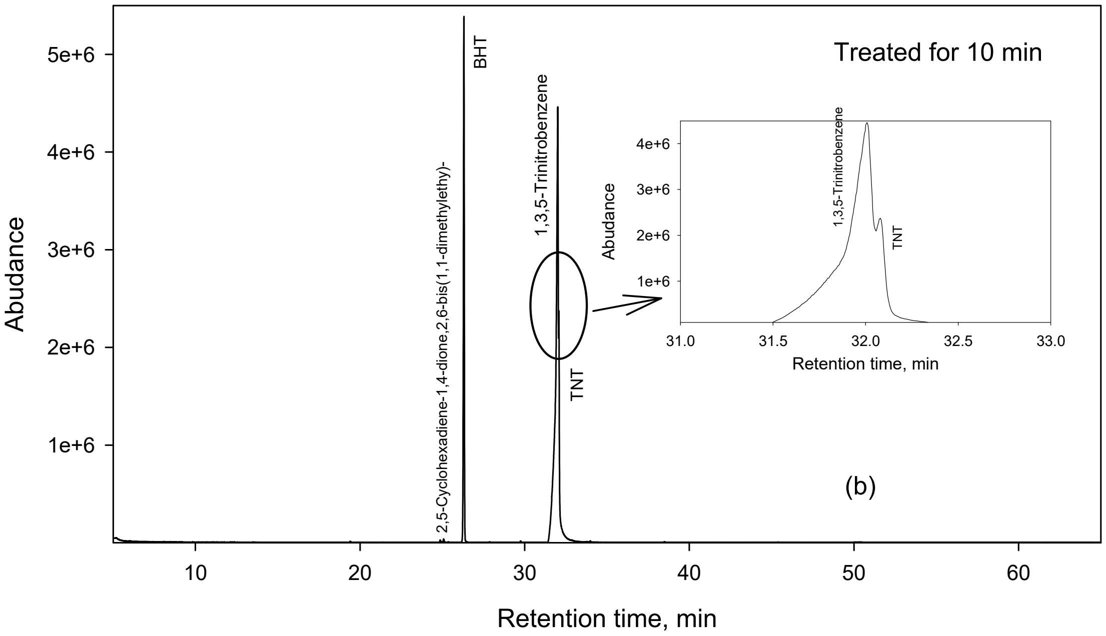
Treated for 10 min
(b)
Abudance
Retention time, min
2,5-Cyclohexadiene-1,4-dione,2,6-bis(1,1-dimethylethy)-
BHT
1,3,5-Trinitrobenzene
TNT

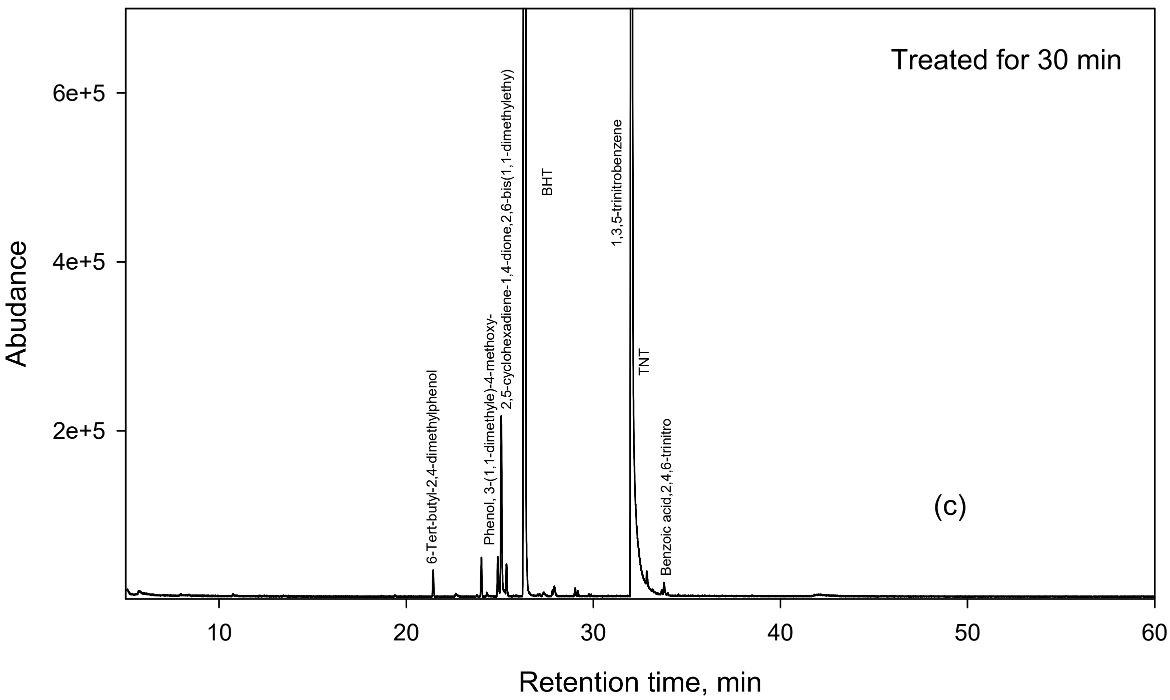
Treated for 30 min
Abudance
Retention time, min
6-Tert-butyl-2,4-dimethylphenol
Phenol, 3-(1,1-dimethyle)-4-methoxy-
2,5-cyclohexadiene-1,4-dione,2,6-bis(1,1-dimethylethy)
BHT
1,3,5-trinitrobenzene
TNT
Benzoic acid,2,4,6-trinitro
(c)

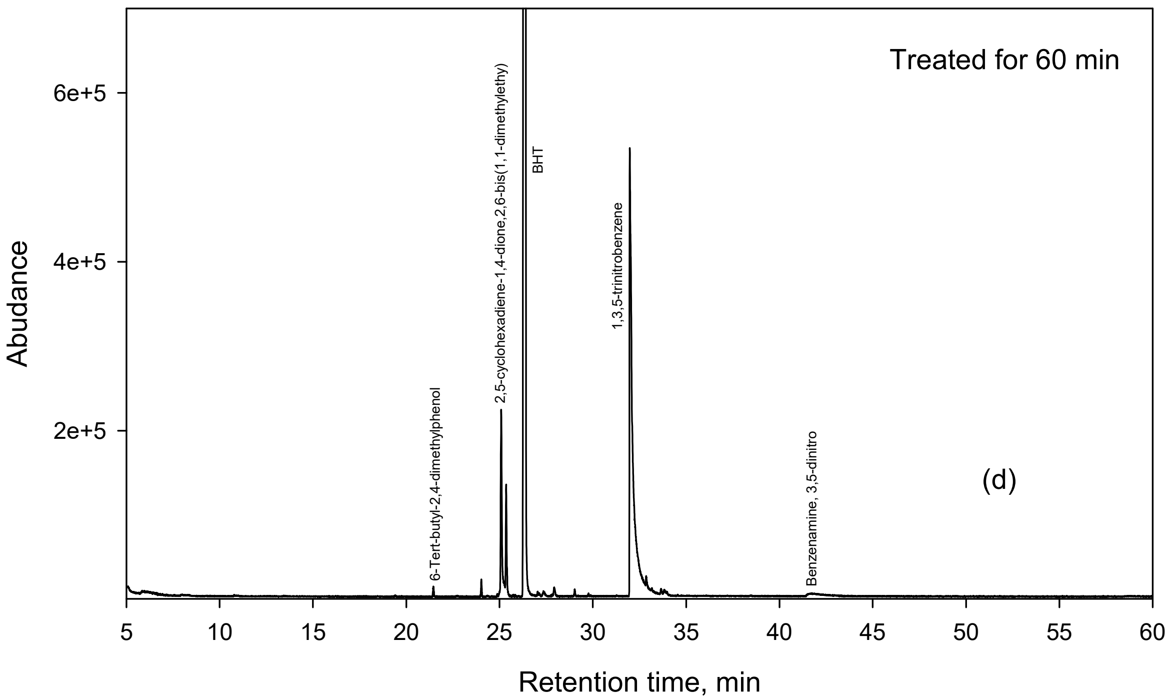
Treated for 60 min
Abudance
Retention time, min
6-Tert-butyl-2,4-dimethylphenol
2,5-cyclohexadiene-1,4-dione,2,6-bis(1,1-dimethylethy)
BHT
1,3,5-trinitrobenzene
Benzenamine, 3,5-dinitro
(d)

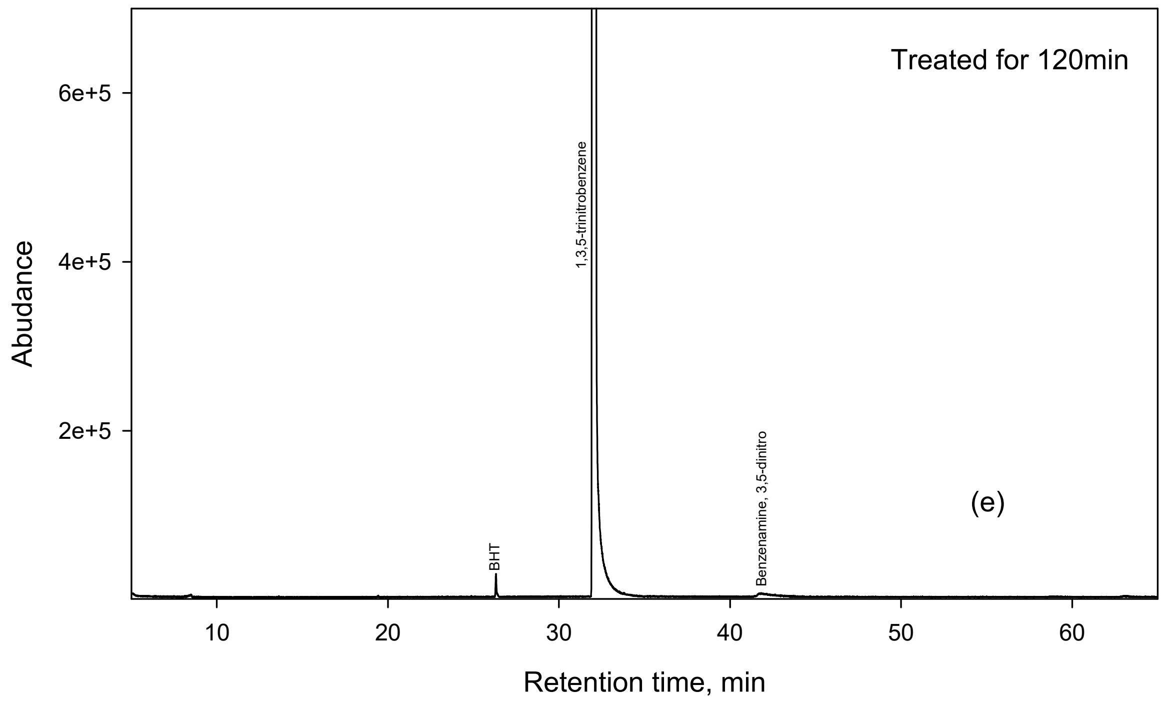

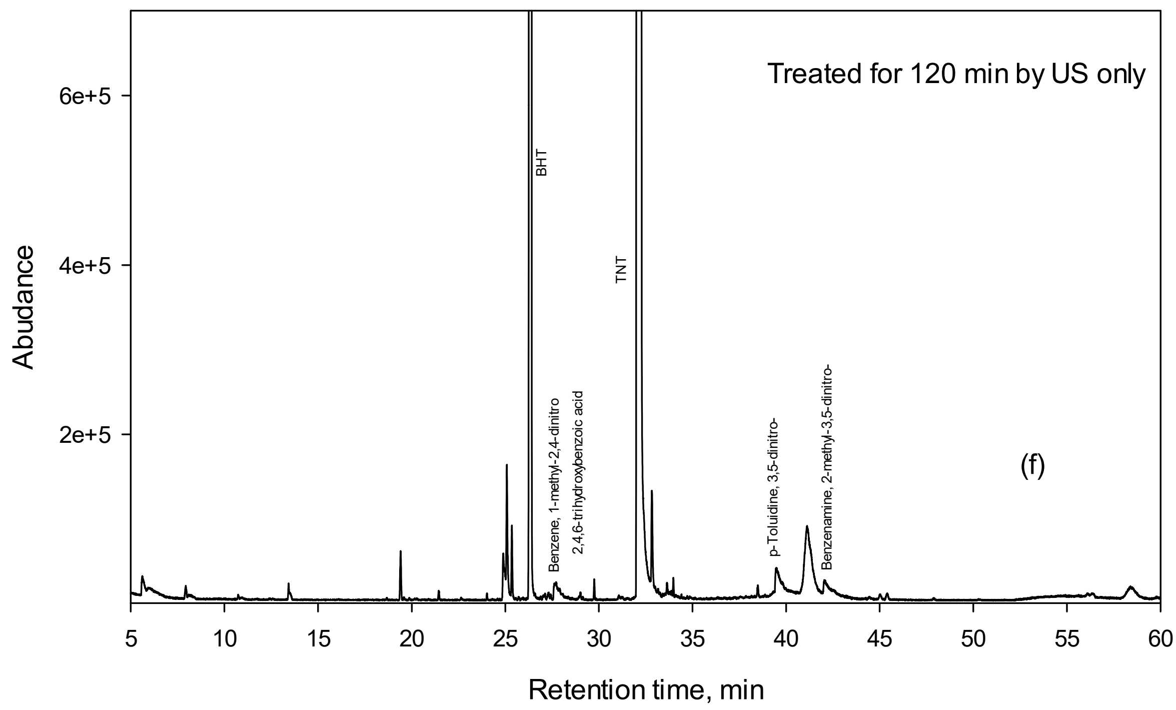

Figure S1 GC/MS profiles of the extract of wastewater treated for different time and the main intermediates. (a): original TNT sample, (b): TNT sample treated for 10 min, (c): TNT sample treated for 30min, (d): TNT sample treated for 60min, (e): TNT sample treated for 120 min. (f): TNT sample treated for 120 min by US only. Experimental conditions: $[TNT]_0$ = 30 mg L^{-1}, [NaCl] = 10^{-2} M, Temperature = 25℃, $[Fe^{2+}]$ = 5×10^{-4}M, $[H_2O_2]$ = 5×10^{-3}M, Initial pH = 3.0 ± 0.1, US intensity = 300 watts cm^{-2}, Reaction time = 0, 10, 30, 60 ,120min.

Reference

1. Huang, C.P.; Dong, C.; Tang, Z. Advanced chemical oxidation: Its present role and potential future in hazardous waste treatment. Waste Management, 1993,13, 361-377. https://doi.org/10.1016/0956-053X(93)90070-D
